# 

**Published:** 1871-01

**Authors:** 


### PERIODICALS AND BOOKS.

HERALD OF HEALTH.—A valuable health journal, published by Wood & Holbrook, New York, at $2.00 a year. Furnished with the BISTOURY at the same price.

LITTLE CORPORAL.—The best juvenile monthly in America. One that we can gladly recommend to every family. Sewell & Miller, Publishers, Chicago, Ill. Subscription price $1.50. Clubs with the BISTOURY at same price.

GOOD HEALTH.—A monthly journal of physical and mental culture, published by Alex. Moore, 11 Broomfield St., Boston, at $2.00 a year. Almost everything in the book line eminating from Boston, is good. "Good Health" is no exception. We will furnish it with the BISTOURY for $2.00 a year.

THE MEDICAL TIMES.—A high-toned, semi-monthly medical journal, published by J. B. Lippincott & Co., Phil., at four dollars a year. It contains original Lectures and communications from the best physicians of our land, as well as clinical reports from our principal Hospitals. It is the best printed medical journal published. Send 20 cts. to the publishers for a sample copy.

SCIENTIFC AMERICAN.—This sterling, scientific monthly is in its 23d volume, and continues to furnish valuable information to the mechanic, farmer, student, and all classes of men. It is finely illustrated and beautifully printed. Price $3.00 a year. Munn & Co., 37 Park Row, New York, Publishers. We will cause it to be sent to any addrcss, together with the BISTOURY upon receipt of price.

OUR SEVEN CHURCHES. THOS. K. BEECHER, ELMIRA, N. Y., 1870. FOR SALE BY HALL BROTHERS. PRICE $1.00.

The book is made up of seven most excellent lectures, recently delivered in the Opera House, in this city, upon the *good* existing in the Roman Catholic, Presbyterian, Protestant Episcopal, Baptist, Congregational and Liberal Christian Churches; together with two other lectures, one upon Choosing one's Church, and another upon "The Church of Christ." The little book has met with a very large sale, and should be read by every church-going person.

THE NATIONAL WORK.--WEBSTER'S UNABRIDGED DICTIONARY, just published, revised, enlarged and handsomely embellished, is one of the most wonderful works of the day. It is a library in itself, by reason of its fulness of definition in matters of Art and Science. It is a magnificent, large, well bound, and extensively illustrated volume, of about 1,800 pages, and is sold by all booksellers, at $12.00. Sent to any address, upon recept of price, by Hall Brothers, or furnished for fifty subscribers to the BISTOURY.

A FOURTEEN WEEKS COURSE IN POPULAR GEOLOGY. J. DORMAN STEELE, A. M., Ph. D. A. S. BARNES & CO., PUBLISHERS, NEW YORK, 1871.

This is a companion book to Prof. Steele's Fourteen weeks in Chemistry, Astronomy, and Natural Philosophy; and like its companions, is a plainly and entertainingly written treatise, giving the outlines of the science without dipping too freely into the technicalities and obstruse theories of geology, confusing and discouraging the young mind on the threshold of his subject. Prof. Steele knows how to write entertaining as well as instructive school books, better than any teacher we know of. His books are meeting with an immense sale, as they deserve to.

PALACE & HOVEL: OR PHASES OF LONDON LIFE.—A large and beautifully illustrated volume, written by Daniel Joseph Kirwan, the able journalist, who has spent much of his time in and about London, gathering authentic materials for his book. He exhibits London life in all its phases—introduces you to the palaces of the nobility, and carries you to the underground hovels of the gutter snipes; illustrates the every day life of the Royal Family, and other notables, giving many facts and incidents not known to the public. It is a highly interesting book, giving you more information of London than you could obtain by a year's sojourn in that great city. BELKNAP & BLISS, Publishers, Hartford, Conn. Sold only by subscription.—Agents wanted.

THE PHARMACIST.—A monthly journal, devoted to Pharmacy, Chemistry, and the Collateral Sciences. Published by the "Chicago College of Pharmacy," Chicago, Ill. One dollar a year.

THE YOUNG FOLKS' RURAL is an eight page paper, large, handsomely illustrated, and designed to cultivate a taste for Rural life. Published by H. N. F. Lewis, Chicago, Ill. Price $1.00 a year.

### Answers to Correspondents.

L. A. B.—*Honesdale, Pa.*—We don't know him, neither do we wish to. Stear clear of such charlatans.

MRS. H.—*Hornbrook, Pa.*—Send in the names of your subscribers, with the money, as fast as you secure them. We will give you credit on our Club Book, and send you the sewing machine, or any of our other premiums, as soon as the club is completed.

L. R. S.—*Richburg, Va.*—Do not apply the plaster to the cancer. It is a very dangerous experiment. If you have it removed, apply to some surgeon, and not to a Cancer Quack.

DR. B.—*Adrian, Mich.*—Holmes' Surgery is best for your purpose. It is more thorough. There are five volumes, costing $9.50 a volume,

MRS.——*Athens, Pa.*—No doubt your trouble is caused by the pills taken. We have seen several just such cases, from the same cause. Better let nature take its course. Your excuse is not a sufficient one.

DR. R. B.—*Hughesville, Ohio* —The pills are too highly extolled, as is apt to be the case with nine-tenths of the proprietary medicines, prepared by wholesale druggists, nowadays. They are made to sell. We have no confidence in them.

MRS. Y. M.—*Spring Valley, Miss.*—Your husband is a brute. You would be justifiable in leaving him. There are very many women who suffer equally with you.

DR. R.—*Northwood, Iowa.*—Much obliged for your club, and kind words. Dr. Sheldon's truss is the very best in the market. You will find his address in the BISTOURY.

DR. A. T.—*Joe's Bayou, La.*—You can secure the instrument of Geo. Triemann & Co., 67 Chatham St., N. Y.

DR. N.—*Cokerville, Ala,*—We do not know of any such work. The medicines, we suppose, can be had of any druggist in New York City.

DRUGGIST.—*Owego, N. Y.*—You can bleach lard by adding a very small proportion of a mixture of bichromate of potassa and muriatic acid to it.

J. R. M.—*Hornellsville, N. Y.*—You will find a recipe among the "medical items and news," in this number of the BISTOURY, that will inform you how to prepare anatomical specimens.

MRS. H.—*Troy, Pa.*—Have the bunyon opened ; press the matter all out, and then have the sac injected with tincture of iodine. This treatment will destroy the sac and prevent the flud from reforming.

DR. A.—*Troy, Pa.*—You can procure Dr. Babcock's Uterine Supporter in this city. See advertisement in BISTOURY.

